# Modification of Soybean 11S Protein by Fermentation: Antioxidant Capacity, Oxidative Stability in Emulsions and Structural Evolution

**DOI:** 10.3390/foods15020199

**Published:** 2026-01-07

**Authors:** Yaozu Guo, Jiaxuan Han, Boxing Yin, Ruixia Gu, Dawei Chen, Zhangwei He, Congcong Tang, Wenqiong Wang

**Affiliations:** 1 College of Food Science and Engineering, Yangzhou University, Yangzhou 225127, China; gyz15240477626@163.com (Y.G.); hanjiaxuan202@163.com (J.H.);; 2 Shaanxi Key Laboratory of Environmental Engineering, School of Environmental and Municipal Engineering, Xi’an University of Architecture and Technology, Xi’an 710055, China

**Keywords:** soybean 11S protein, fermentation, antioxidant capacity, oxidative stability, conformational change

## Abstract

Fermentation is an effective method to enhance the bioactivity of plant proteins, yet the link between the functionality and conformational state of fermented soybean 11S protein (F11S) requires clarification. This study first evaluated the antioxidative efficacy of F11S and its application in emulsion systems, followed by a mechanistic investigation into its structural evolution. Results showed that the bioactivity of F11S was strictly fermentation-time-dependent, reaching its peak at 16 h. At this stage, F11S exhibited maximal scavenging capacities for ·OH (84.51 ± 2.53%) and DPPH radicals (93.84 ± 2.62%). Crucially, in a Tween 20 emulsion system, the F11S-16h fraction demonstrated superior oxidative stability, maintaining the lowest peroxide value (4.33 ± 0.53 mmol/kg) after 15 days of storage. To elucidate the mechanism behind this enhanced functionality, structural analysis was conducted. It revealed that while surface hydrophobicity peaked at 12 h due to protein unfolding, extended fermentation to 16 h induced a refolding process, guiding the protein into a thermodynamically stable conformation. These findings indicate that the stable refolded structure formed at 16 h, rather than maximal hydrophobicity, is the key determinant for the superior antioxidant performance and emulsion stabilizing ability of F11S.

## 1. Introduction

Soybean protein is widely recognized as a premium plant-based protein source due to its balanced amino acid profile and functional versatility [1]. Among its components, 11S globulin (11S/glycinin) accounts for approximately 30–40% of the total protein and plays a dominant role in determining the texture and stability of soy-based food systems [2]. However, the application of native 11S in lipid-rich formulations (e.g., emulsions) is often limited. Native 11S is susceptible to environmental stresses and often lacks sufficient interfacial activity to prevent lipid oxidation, a primary cause of off-flavors and nutritional degradation in food products [3]. Therefore, structural modification strategies to enhance the antioxidative potential and emulsifying stability of 11S globulin are of significant research interest. Lactic acid bacteria (LAB) fermentation has emerged as a safe and effective biological modification strategy [4]. Compared to chemical treatments, fermentation can gently modify protein structures and release bioactive components [5]. The previous study has documented that fermentation enhances the antioxidant capacity of soy matrices. For instance, bioconversion of isoflavone glycosides into aglycones during fermentation has been shown to effectively scavenge free radicals [6]. Similarly, selective proteolysis by microbial enzymes can release bioactive peptides with high antioxidative activity [7]. Consequently, fermented soy products typically exhibit superior bioactivity compared to their unfermented counterparts.

However, a critical knowledge gap remains regarding the macromolecular evolution of the 11S protein itself. While previous studies have heavily focused on small-molecule metabolites (e.g., isoflavones, peptides), the conformational dynamics of the 11S during the fermentation process—specifically its unfolding and subsequent refolding behaviors—have been largely overlooked [8,9]. It remains unclear how these fermentation-induced structural changes (e.g., surface hydrophobicity, secondary structure reorganization) directly correlate with the protein’s ability to inhibit lipid oxidation in complex emulsion systems. Understanding this structure–function relationship is crucial for rationalizing the use of fermented proteins as functional ingredients.

To address this, this study investigates the dynamic evolution of fermented 11S (F11S) over varying fermentation times. The specific objectives are to (1) evaluate its in vitro antioxidant capacity; (2) determine the oxidative stability of F11S-stabilized emulsions (specifically against lipid thermal oxidation); and (3) characterize the conformational changes of F11S, focusing on the interplay between the structure of F11S and its antioxidant function. This work aims to elucidate the mechanism by which specific structural states confer superior antioxidative functionality, providing a theoretical basis for the application of F11S in functional food formulations.

## 2. Materials and Methods

### 2.1. Materials

Soybeans were commercially sourced from Tmall supermarkets (Yangzhou, Jiangsu, China). Culture medium MRS broth was purchased from Qingdao Hi-Tech Industrial Park Haibo Biotechnology Co. (Qingdao, China). The lactic acid bacteria (LAB) strains *Limosilactobacillus fermentum* 56 (Lf. 56) and *Limosilactobacillus fermentum* 57 (Lf. 57) were acquired from the Key Lab of Dairy Biotechnology and Safety Control, Yangzhou University.

### 2.2. 11S Protein from Soybean Preparation

Soybean 11S protein was isolated following the extraction protocol established by Nagano et al. [10], Liu et al. [11] and Gao et al. [12] with slight modifications. Soybeans were pulverized to achieve a homogeneous powder and sieved through an 80-mesh sieve (0.2 mm aperture). The powder was degreased with a mixture of hexane and 95% ethanol (*w*:*v*:*v* 1:3.6:0.4) and agitated at room temperature. After filtering every 2 h until the solution became clear, the defatted powder was dried at room temperature. The defatted powder was then mixed with deionized water (1:15 *w*:*v*) and adjusted to pH 8.5. After stirring for 1 h, the mixture was centrifuged at 4 °C, 9000× *g* for 30 min. The supernatant was collected, NaHCO_3_ was added to a final concentration of 0.98 g/L, and the pH was adjusted to 6.4. The solution was left to stand overnight at 4 °C, then centrifuged at 4 °C, 6000× *g* for 30 min. The precipitate was washed three times with deionized water, re-dispersed in deionized water (*w*:*v* 1:5), and adjusted to pH 7.0. Finally, the solution was freeze-dried to obtain soybean 11S protein (11S) powder and stored at −20 °C.

### 2.3. Fermented Soybean 11S Protein (F11S) Preparation

The 11S protein was dissolved in deionized water to a concentration of 3% (*w*/*v*), and glucose (2%, *w*/*v*) was added to the solution as a carbon source for LAB growth. [13]. The pH of the solution was adjusted to 7.0 using 2 M HCl, and then it was sterilized by heating at 85 °C for 10 min. The second-generation activated LAB strains Lf. 56 and Lf. 57 were inoculated into the sterilized solution above at an inoculum size of 5% (*v*:*v*). And the solution was hatched at 37 °C for 20 h to obtain F11S. Samples were collected at 0, 8, 12, 16, and 20 h for the following studies.

### 2.4. Inhibition of Lipid Peroxidation Induced by Iron

The liposome PBS dispersion system (LLS) was prepared following the method of Gu et al. [14]. Briefly, 30 mg of lecithin was dissolved in 30 mL of 10 mmol/L PBS solution (pH 7.4) and subjected to ultrasonication to obtain a homogeneous liposome dispersion. In a sample tube, 1 mL of LLS, 1 mL of 400 μmol/L ferrous sulphate solution, and 1 mL of the sample were sequentially added and mixed thoroughly. The mixture was incubated in the dark at 37 °C in a water bath for 60 min, followed by the addition of 2 mL of trichloroacetic acid (TCA)-thiobarbituric acid (TBA)-hydrochloric acid (HCl) mixture (TCA-TBA-HCl mixture: prepared by dissolving 15 g TCA, 0.375 g TBA, and 2.1 mL HCl sequentially in 100 mL water). The reaction was incubated in a 100 °C water bath for 15 min, cooled, and then centrifuged at 5500 r/min for 10 min. The absorbance of the supernatant was measured at 532 nm.


(1) $$\mathrm{Elimination}\;\mathrm{rate}/\%=\left(1-\frac{A_{s}-A_{c}}{A_{c}}\right)\times 100,$$

In the formula, A_s_ represents the absorbance of the sample tube, and A_c_ represents the absorbance of the blank tube, where water replaced the sample solution.

### 2.5. ·OH Radical Scavenging Activity Assay

The ·OH radical scavenging activity was assayed following the method of Shen et al. [15]. A mixture was prepared by adding 1.00 mL of sample solution (30 mg/mL), 1.00 mL of ferrous sulphate solution (10 mmol/L), and 1.00 mL of salicylic acid-ethanol solution (10 mmol/L) to a test tube. Subsequently, 1.00 mL of hydrogen peroxide (0.03%) was added to initiate the reaction. The mixture was shaken and incubated in a water bath at 37 °C for 30 min, followed by centrifugation at 5500 r/min for 7 min. The absorbance was measured at 510 nm.

(2) $$\mathrm{Elimination}\;\mathrm{rate}/\%=\left(1-\frac{A_{s}-A_{0}}{A_{c}-{\;A}_{0}}\right)\times 100,$$

In the formula, A_c_ represents the absorbance of the sample without the blanking agent, A_s_ represents the absorbance of the sample solution with the blanking agent, and A_0_ represents the absorbance of the blank tube, where water replaced the sample.

### 2.6. DPPH Radical Scavenging Activity Assay

The DPPH radical scavenging activity was determined following the method of Brand-Williams et al. [16]. The sample was diluted to a concentration of 30 mg/mL. Prior to mixing, the F11S samples were fully dispersed in deionized water to ensure solubility. Subsequently, 1.0 mL of the sample was mixed with 4.0 mL of 0.12 mmol/L DPPH ethanol solution (95% *v*:*v*). The mixture was vortexed thoroughly and incubated at room temperature in the dark for 30 min. The reaction solution was then centrifuged at 5500 r/min for 5 min. Using a 95% ethanol solution as the reference, the absorbance was measured at 517 nm. The scavenging rate was calculated according to the equation.

(3) $$\mathrm{Elimination}\;\mathrm{rate}/\%=\left(1-\frac{A_{i}-A_{j}}{A_{c}}\right)\times 100,$$

In the formula, A_i_ represents the absorbance of the DPPH solution after adding the sample solution, A_j_ represents the absorbance of the sample solution, and A_c_ represents the absorbance of the DPPH solution without adding the sample solution.

### 2.7. Preparation and Analysis of F11S Emulsion

Following the method of Cheng Yu et al., soybean oil was mixed with 0.1 M phosphate buffer (pH 7.0) at a weight ratio of 10:90, with 10 mg/mL Tween 20 added concurrently [17]. The mixture was stirred at 25 °C for 10 min using a magnetic stirrer, then homogenized twice through a high-pressure (36 MPa) valve homogeniser to obtain a fine emulsion. Add F11S at different time points to the emulsion (F11S: emulsion 1:2 *v*/*v*).

The Schaal oven method was employed to evaluate the effect of F11S on combating lipid thermal oxidation during accelerated oil storage [18]. Sample preparation was designated as day 0. Following placement in the oven, samples were stirred once daily and sampled every three days. An emulsion sample of unfermented F11S served as the control, while a 0.01% *w*/*w* BHA solution constituted the control group.

### 2.8. Peroxide Value (POV) and Acid Value (AV) Analysis

The POV and AV were measured to assess the oxidative stability and acidity of the F11S emulsion, providing insights into the emulsion’s susceptibility to lipid oxidation during storage. POV and AV were measured following the method by Wang et al. with slight modification and in this study, the method was performed according to the standard of GB 5009.227-2023 and GB 5009.229-2025 [19].

The POV was determined based on the iodometric titration method. Approximately 1–2 g of the F11S emulsion sample was accurately weighed into a dry 250 mL iodine flask. The sample was dissolved in 30 mL of an isooctane/glacial acetic acid mixture (2:3, *v*:*v*) by gentle swirling. Subsequently, 1 mL of saturated potassium iodide (KI) solution was added; the flask was stoppered, shaken, and incubated in the dark for 3 min. The reaction was immediately halted by the addition of 50 mL of distilled water. The mixture was then titrated with 0.01 mol/L sodium thiosulfate (Na_2_S_2_O_3_) standard solution. When the aqueous layer turned pale yellow, 1 mL of starch indicator was added, and the titration continued until the blue color completely disappeared (recorded as V_1_). A blank determination was performed simultaneously under the same conditions without the oil sample (recorded as V_2_). The POV was calculated according to the equation.

(4) $$\mathrm{POV}\;(\mathrm{mmol}/\mathrm{kg})=\frac{C\times \left(V_{1}-V_{2}\right)\times 1000}{W},$$

In the formula, V_1_ and V_2_ represent the volumes (mL) of Na_2_S_2_O_3_ consumed by the sample and the blank, respectively, *c* is the concentration of the standard solution (mol/L), and W is the mass of the oil sample (g).

The AV was determined according to the following procedure. F11S emulsion samples (3–4 g) were placed into 250 mL Erlenmeyer flasks. Each sample was dissolved in 50 mL of a neutralized diethyl ether/ethanol mixture (2:1, *v*:*v*) by gentle swirling. After the addition of 2–3 drops of phenolphthalein indicator, the solution was titrated with 0.1 mol/L ethanolic potassium hydroxide (KOH). The titration was continued until a faint pink color persisted for at least 30 s. The volume of the titrant consumed was recorded as V. The AV was calculated according to the equation.

(5) $$\mathrm{AV}\;(\mathrm{mg})=\frac{N\times V\times 56.1}{W}$$

In the formula: V is the volume of standard KOH solution consumed (mL). N is the concentration of the KOH solution (mol/L). 56.1 is the molar mass of KOH (g/mol). W is the mass of the F11S emulsion samples (g).

### 2.9. Intrinsic Fluorescence Measurements

Intrinsic fluorescence was used to investigate the structural characteristics and conformational changes of F11S and unfermented 11S. Intrinsic fluorescence was measured following the method by Gao et al. using a spectrofluorometer (Shimadzu RF-5301PC, Tokyo, Japan) [12]. The fluorescence of 0.06 mg/mL solutions of F11S and unfermented 11S was recorded at room temperature with a 10 mm path length. The excitation wavelength was set at 290 nm, and emission spectra were recorded within the range of 300 to 400 nm with both excitation and emission slits set to 5 nm.

### 2.10. Circular Dichroism (CD) Measurements

CD measurements were employed to evaluate the secondary and tertiary structures of F11Sand unfermented 11S. CD measurements were performed as described by Sui et al. with slight modifications, using a spectrofluorometer (Applied Photophysics J-810, JASCO, Hachioji, Japan) [20]. Changes in protein secondary structure were determined using far-UV CD (190–260 nm) with 3 mg/mL solutions of F11S and unfermented soybean 11S protein with a 2 mm path length. The tertiary structure was assessed by near-UV CD (250–310 nm) with a 10 mm path length. Three scans of the above at room temperature.

### 2.11. Surface Hydrophobicity Measurements

Surface hydrophobicity was determined using the ANS fluorescence probe to understand the exposure of hydrophobic regions in F11S and unfermented 11S. Following the method of Zhu et al., surface hydrophobicity was determined using the 8-anilino-1-naphthalenesulfonic acid (ANS) fluorescence probe [21]. Fluorescence measurements were performed on a spectrofluorometer (Shimadzu RF-5301PC, Tokyo, Japan). To 3 mL of 0.5 mg/mL F11S and unfermented soybean 11S protein solutions, 50 μL of 8 mM ANS solution was added. The measurements were conducted with a 1 cm path length, excitation at 390 nm, and emission recorded from 400 to 600 nm, with both slits set to 5 nm.

### 2.12. Statistical Analysis

The tests were repeated three times and all analyses were performed in triplicate. Experiments were conducted in a randomized factorial design, and the data obtained were analyzed using SPSS ver.19 software. Results are expressed as mean ± standard error. Duncan’s multi-range test was utilized at 0.05 significance (*p* < 0.05) to compare the averages of the test groups. Duncan’s test was also used to compare the average scores of judges on the 5-point hedonic scale.

## 3. Results and Discussion

### 3.1. Inhibition of Lipid Peroxidation of Fermented Soybean 11S Protein (F11S)

**Figure 1:**
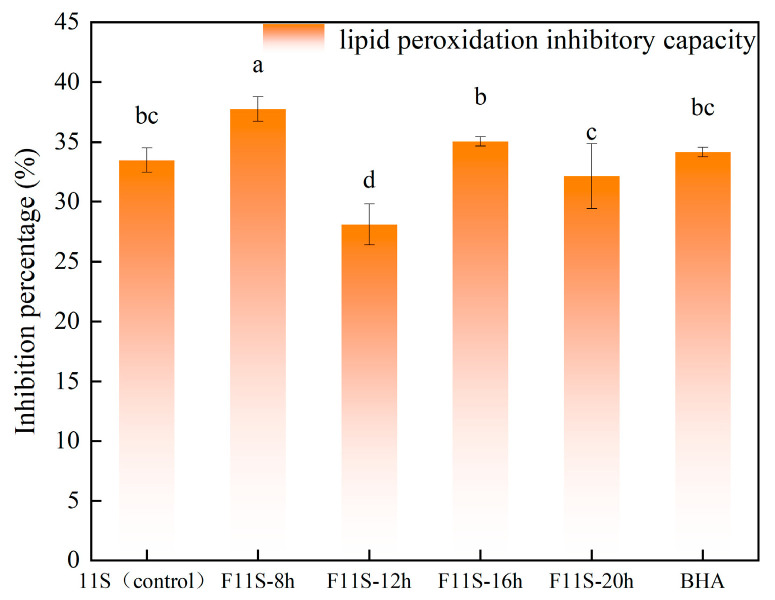
The lipid peroxidation inhibitory capacity of F11S at different fermentation times and 0.01% BHA. Different lowercase letters indicate a significant difference between lipid peroxidation inhibitory capacity (*p* < 0.05).

Before evaluating the bioactivity, the fundamental characteristic of the fermentation process was monitored. The pH of the fermentation broth decreased significantly from an initial 7.0 to 3.25 ± 0.1 after 20 h, indicating the vigorous metabolic activity of the LAB strains and the accumulation of organic acids. The lipid peroxidation inhibitory capacity of fermented soybean 11S (F11S) at different fermentation times and 0.01% BHA is shown in Figure 1, alongside unfermented soybean 11S (11S) and 0.01% BHA as controls. Significant differences (*p* < 0.05) were observed among these groups. F11S fermented for 8 h and 16 h demonstrated enhanced lipid peroxidation inhibition, reaching 37.75 ± 1.03% and 35.06 ± 0.4%, respectively, showing an increase of 4.27% and 1.57% compared to the unfermented 11S group, which exhibited an inhibition of 33.48 ± 1.03%. However, at fermentation times of 12 h and 20 h, F11S exhibited lower inhibition percentages (28.09 ± 1.7% and 32.13 ± 2.7%) showing decreases of 5.39% and 1.35%, respectively, compared to the unfermented 11S. These results suggest that while fermentation can enhance antioxidant activity, possibly by generating compounds that inhibit the propagation step of oxidative chain reactions [22], prolonged or specific fermentation durations might lead to the degradation of active compounds or the formation of less effective forms.

The differing characteristics of chelating ferrous ions may either delay or accelerate lipid oxidation by either eliminating or enhancing the pro-oxidative activity of ferrous ions [23]. Therefore, further measurements were conducted on the F11S ability to scavenge ·OH radicals and its capacity to scavenge DPPH radicals.

### 3.2. ·OH Radical Scavenging Activity of F11S

**Figure 2:**
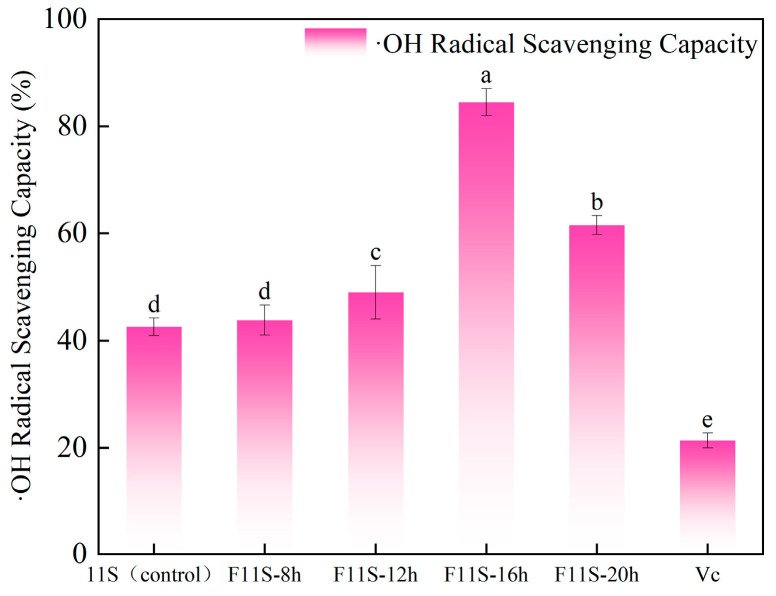
The ·OH scavenging capacity of F11S at different fermentation times and 0.01% vitamin C (Vc). Different lowercase letters indicate a significant difference between ·OH scavenging capacity (*p* < 0.05).

After lactic acid bacteria (LAB) fermentation, the ·OH scavenging capacity of F11S at different fermentation times and 0.01% vitamin C (Vc) is shown in Figure 2. Significant differences (*p* < 0.05) were observed among all groups. Notably, both unfermented 11S protein and F11S at all tested fermentation stages exhibited markedly superior ·OH scavenging capacities compared to 0.01% Vc. Following LAB fermentation, the ·OH scavenging capacity of F11S significantly increased, progressively enhancing with extended fermentation duration to peak at 84.51 ± 2.53% at 16 h, showing an increase of 41.94% compared to the unfermented 11S group (42.57 ± 1.65%). However, a subsequent decline was observed by 20 h of fermentation, reaching 61.48 ± 1.77%, showing a decrease of 21.18% from the peak at 16 h. This non-linear relationship suggests that optimal fermentation duration is crucial for maximizing ·OH scavenging activity. The initial enhancement is likely attributable to altered protein conformation during fermentation, which may expose or generate new functional groups capable of effectively scavenging ·OH radicals. The observed reduction in ·OH scavenging capacity of F11S at 20 h could stem from further conformational changes, degradation, or aggregation [24,25].

### 3.3. DPPH Radical Scavenging Activity of F11S

**Figure 3:**
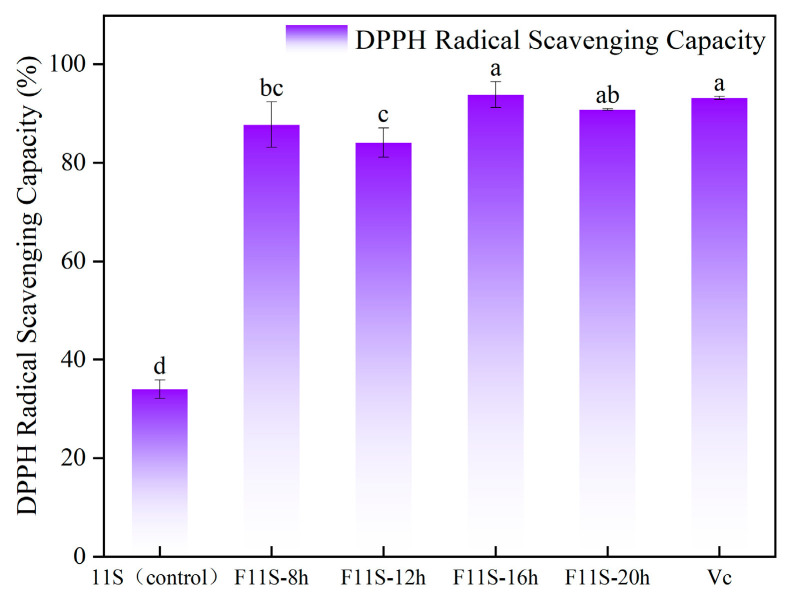
The DPPH scavenging capacity of F11S at different fermentation times and 0.01% vitamin C (Vc). Different lowercase letters indicate a significant difference between DPPH scavenging capacity (*p* < 0.05).

The DPPH radical scavenging capacity of F11S at various fermentation time points, compared to unfermented 11S protein and 0.01% Vc is shown in Figure 3. Significant differences (*p* < 0.05) were observed among all groups. LAB fermentation significantly enhanced the DPPH scavenging capacity of 11S (*p* < 0.05). F11S exhibited markedly higher activity than unfermented 11S at all fermentation durations. The activity peaked at 93.84 ± 2.62% at 16 h of fermentation, showing a 30.11% increase compared to the unfermented 11S (34 ± 1.88%). This substantial improvement is primarily attributable to the degradation of soybean 11S protein into low molecular weight peptides by LAB during fermentation [26].

### 3.4. Peroxide Value (POV) of F11S Emulsion

Oil-in-water (O/W) emulsions are prevalent in food products, including beverages, sauces, and soups [27]. However, their susceptibility to oxidation presents a significant challenge to product stability [28]. To counteract this degradation, antioxidants are commonly incorporated. Natural antioxidants are particularly favored due to their superior safety profiles. Among these, protein hydrolysates have emerged as a novel and promising class of antioxidants owing to their broad free radical scavenging capabilities [29].

**Figure 4:**
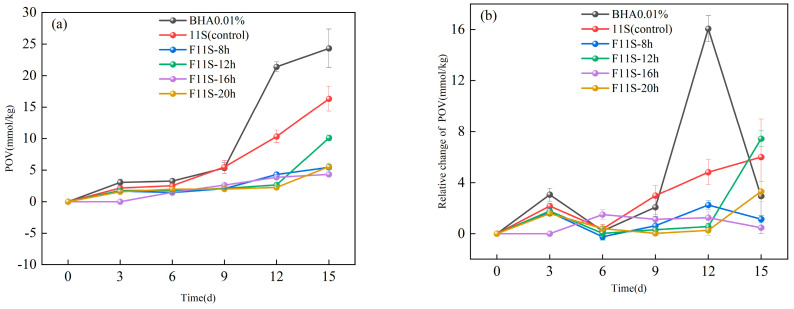
The POV of the F11S emulsion and a 0.01% BHA: (**a**) The changes in POV for the F11S emulsion and a 0.01% BHA; (**b**) The relative changes in POV during storage for F11S emulsions and 0.01% BHA.

To mitigate flocculation and enhance the stability of F11S emulsions during storage, 10 mg/mL Tween 20 was incorporated. The POV reflects the hydrogen peroxide generated during the initial stage of lipid oxidation. Figure 4a illustrates the changes in POV for the F11S emulsion and a 0.01% BHA solution across different storage periods.

F11S exerted negligible influence on peroxide formation during the initial storage phase (0–6 days). However, after 9 days of storage, peroxide formation was significantly inhibited (*p* < 0.05) in emulsions supplemented with F11S. At 15 days of storage, 0.01% BHA exhibited the highest POV (23.33 ± 3.05 mmol/kg), followed by the 11S emulsion (unfermented 11S) (16.32 ± 2 mmol/kg). Conversely, F11S emulsions significantly reduced peroxide values, with F11-16h demonstrating the most potent effect, yielding the lowest value (4.33 ± 0.53 mmol/kg). This finding aligns with our previous observations that F11S exhibited its strongest antioxidant activity after 16 h of fermentation, and is consistent with prior research indicating that protein hydrolysates effectively inhibit lipid oxidation [30].

To further elucidate the antioxidative performance of F11S emulsions, Figure 4b presents the relative changes in POV during storage for F11S emulsions and 0.01% BHA. Throughout the storage period, significant differences (*p* < 0.05) were observed in these relative changes among all samples. Notably, F11S emulsions, regardless of fermentation duration, consistently exhibited lower relative changes in POV compared to emulsions containing either 11S emulsion or 0.01% BHA at all storage periods, with the exception of 6 d. The 0.01% BHA showed the highest relative change in POV at 3 d (3.50 ± 0.51 mmol/kg) and 12 days (16.06 ± 1.02 mmol/kg). However, at 15 d, this relative change decreased to 2.93 ± 2.9 mmol/kg. This apparent reduction in the rate of peroxide accumulation might be due to hydrogen peroxide saturation as emulsion oxidation progressed [31].

For native 11S emulsion, while a slight downward trend was observed at 6 d, from that point onwards, the relative change in POV exhibited a continuous upward trend as the storage time increased. This suggests that the oxidation of the unfermented 11S protein itself likely contributes over time, accelerating hydrogen peroxide production and overall emulsion oxidation.

For F11S emulsions, a similar trend was observed: a significant increase in relative peroxide value at 3 days, followed by a notable decrease at 6 days. Subsequently, with extended storage, these samples showed a gradual increase in the relative changes in POV. This complex behavior, despite these F11S emulsions demonstrating stronger antioxidant activity than their unfermented state (as described in Section 3.1, Section 3.2 and Section 3.3), might reflect the slow oxidation or depletion of the highly antioxidant protein hydrolysates themselves over prolonged storage.

Crucially, F11S-16h emulsion exhibited a significantly different (*p* < 0.05). It displayed a relatively stable relative change in POV throughout the entire storage period, indicating its exceptional ability to suppress lipid oxidation. In conclusion, these findings consistently demonstrate that F11S-16h provides the strongest resistance to lipid thermal oxidation within the O/W emulsion system.

### 3.5. Acid Value (AV) of F11S Emulsions

**Figure 5:**
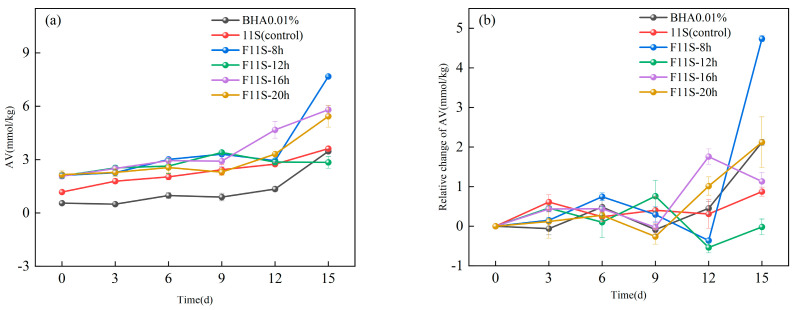
The AV of the F11S emulsion and a 0.01% BHA: (**a**) The changes in AV for the F11S emulsion and a 0.01% BHA; (**b**) The relative changes in AV during storage for F11S emulsions and 0.01% BHA.

AV serves as a crucial indicator of free fatty acid content in oils and fats. Figure 5a illustrates the AV changes of F11S emulsions and 0.01% BHA during storage. As expected, with increasing storage duration, the AV of the emulsions increased significantly (*p* < 0.05) among all samples, primarily due to triglyceride hydrolysis and the generation of free fatty acids. By the 15 d of storage, considerable variation was observed; sample F11S-8h emulsion exhibited the highest AV of 7.68 ± 0.43 mmol/kg. F11S-12h emulsion demonstrated the lowest at 2.85 ± 0.33 mmol/kg at the same time. And for F11S-12h emulsion, AV appeared to gradually decrease from day 9 onwards. This intriguing observation might suggest that F11S-12h could potentially encapsulate lipids during advanced stages of storage, thereby effectively reducing further lipid oxidation and free fatty acid production [32].

During the fermentation of 11S, LAB generate acidic compounds such as lactic or acetic acid. Consequently, F11S emulsions exhibited a higher initial (Day 0) AV compared to native 11S emulsions and the 0.01% w/w BHA solution. However, the absolute initial AV alone does not fully reflect F11S’s ability to affect emulsion acidity or its long-term stability. Therefore, to better assess the dynamic changes, relative changes in AV were analyzed. Figure 5b illustrates these relative changes in AV during storage for F11S emulsions and 0.01% BHA solutions. 0.01% BHA exhibited a slow progression in relative AV change initially, but an accelerated increase was observed at 15 d, reaching 2.12 ± 0.07 mmol/kg. In contrast, the 11S emulsion maintained stable relative AV changes throughout storage, consistently remaining below 1 mmol/kg. Among the F11S emulsions, showed relatively minor changes from 0 d to 12 d. However, at 15 d, F11S-8h emulsion exhibited a sharp increase to 4.74 ± 0.7 mmol/kg, representing the greatest relative change among all samples at this time. This escalation suggests a rapid progression of emulsion oxidation and substantial free fatty acid generation in the F11S-8h emulsion. Conversely, F11S-12h emulsion demonstrated a gradual increase in relative AV change from 0 d to 9 d, followed by a decreasing trend from 12 d. This pattern aligns with the absolute AV data for F11S-12h emulsion presented in Figure 5a. Similarly, both F11S-16h emulsion and F11S-20h emulsion displayed comparable trends. A sharp increase in relative AV change, peaking around 12 d (F11S-16h reaching 1.76 ± 0.19 mmol/kg), followed by a subsequent decrease at 15 d. These varied responses underscore the complex influence of F11S on the dynamic oxidative stability of the emulsions.

In summary, both F11S and unfermented 11S significantly influence the thermal oxidation resistance of emulsions, with F11S generally offering enhanced protection. Specifically, emulsions prepared with F11S-12h notably reduced the formation of free fatty acids, exhibiting the lowest AV.

### 3.6. Tertiary Structure Analysis of F11S

**Figure 6:**
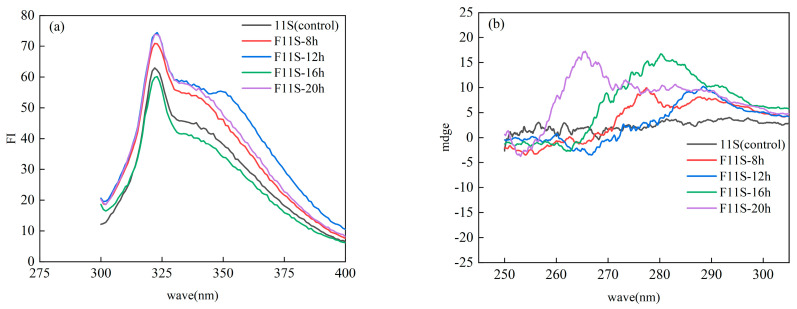
The tertiary structure analysis of F11S: (**a**) The endogenous fluorescence spectra of F11S; (**b**) The near-UV CD spectroscopy for F11S.

The endogenous fluorescence of proteins, primarily attributed to tyrosine (Tyr) and tryptophan (Trp) residues, serves as a crucial indicator of protein polarity and tertiary structural changes [33]. Figure 6a displays the endogenous fluorescence spectra and their maximum absorption wavelengths for F11S. The maximum fluorescence intensity (FI) was observed at 322 nm in the 11S (control, native 11S). This relatively blue-shifted emission wavelength suggests that the Trp residues are predominantly buried within the hydrophobic core of protein.

After fermentation, the FI values of 11S generally increased across various fermentation times compared to the 11S (control) except for F11S-16h. This increase in FI values indicates reduced intramolecular quenching, increased amino acid spacing, and partial exposure of Trp residues within the protein structure [34]. The FI value of F11S-16h was notably lower than that of 11S (control). This observation suggests that at this fermentation duration, there might be increased intramolecular quenching, reduced amino acid spacing, and a more significant encapsulation of Trp residues within the protein structure. Such variations in FI across different fermentation times are indicative of the dynamic nature of molecular conformational expansion and contraction during the process [35].

A red shift in the absorption spectrum maximum was also observed in the F11S. This spectral shift is likely influenced by the decrease in pH attributable to lactic acid and other acidic by-products generated during LAB metabolism. Furthermore, it indicates that fermentation induced partial unfolding of the 11S, thereby shifting some aromatic amino acid residues from a non-polar to a more polar environment [36]. The previous study also suggests that a red shift can arise from Trp residues becoming more deeply buried within the protein core, leading to quenching by neighboring side chains [34]. The overall fluorescence data, particularly the increased FI in most samples alongside the red shift, points towards a complex and dynamic rearrangement of the protein’s tertiary structure rather than a singular change. Consequently, we infer that the 11S adopts a molten-globule state during fermentation. To further assess these complex tertiary structural changes after fermentation, near-ultraviolet circular dichroism (near-UV CD) spectroscopy was subsequently employed.

Near-UV CD spectroscopy (250–310 nm) is a sensitive technique for probing tertiary protein structure [37]. A flat spectral profile typically indicates complete denaturation and a loss of ordered tertiary conformation [12]. As shown in Figure 6b, compared to the unfermented soybean 11S protein, the near-UV CD spectra of F11S exhibited varying degrees of enhancement. This significant alteration and increased signal in the spectral profile of the F11S suggest that LAB fermentation markedly influences and potentially enhances the orderliness of the protein’s tertiary structure.

### 3.7. Secondary Structure Changes of F11S

**Figure 7:**
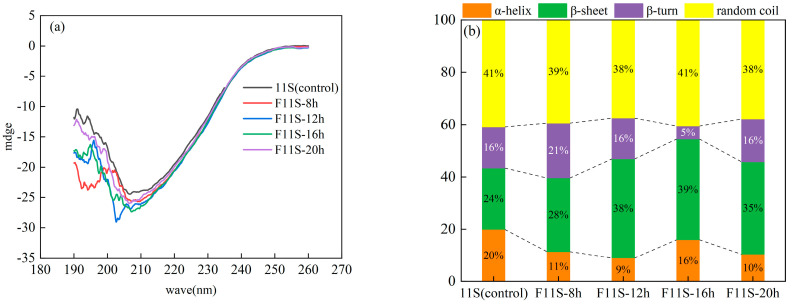
The secondary structure analysis of F11S: (**a**) The far-UV CD spectra of F11S; (**b**) The secondary structure concentration of F11S.

The secondary structure of proteins and peptides is defined by the ordered configurations of their peptide bonds. Far-ultraviolet circular dichroism (far-UV CD) spectroscopy (190–260 nm) serves as an effective method for analyzing and detecting these structural elements [38]. The far-UV CD spectra of 11S and F11S were obtained at various LAB fermentation time points as shown in Figure 7a.

The CD spectrum of 11S exhibited a distinct negative peak at 206.7 nm. Following LAB fermentation, the CD spectrum of F11S underwent significant alterations. The intensity of the main negative peak in the F11S sample was markedly diminished, and the peak itself shifted towards shorter wavelengths (blue shift). Significant reductions in negative ellipticity were observed at 207.3 nm and 206.9 nm in the F11S sample. These spectral changes collectively indicate a partial disruption of the secondary structure of protein due to fermentation. Utilizing the Reed algorithm with CD spectra enables quantitative analysis of α-helix, β-sheet, β-turn, and random coil structures within proteins.

As depicted in Figure 7b, LAB fermentation induced dynamic changes in the secondary structure of F11S protein. Specifically, between 8 and 16 h of fermentation, the α-helix content reaches 9% by 12 h. Conversely, the β-sheet content rose to 39% by 16 h. Simultaneously, the β-turn content fell to 5% by 16 h. The random coil content showed no significant change throughout this period (*p* > 0.05). At 20 h of fermentation, the content of α-helix and β-turn increased once more, and the β-sheet content decreased. These findings suggest that during fermentation, the 11S undergoes a conformational rearrangement, transitioning towards a more ordered or compacted structure by 16 h.

Studies have demonstrated that proteins with a higher β-sheet content typically adopt more compact structures, and enhanced structural stability can indirectly improve antioxidant capacity [39]. In F11S samples, an increased β-sheet content was observed to varying degrees, and the total content of α-helix and β-sheet structures progressively rose after fermentation. Integrating these structural observations with the in vitro antioxidant capacity analyses of F11S at different fermentation times (detailed in Section 3.1, Section 3.2, Section 3.3, Section 3.4 and Section 3.5). We infer that the molten-globule state with higher β-sheet acquired by F11S is instrumental in effectively enhancing the antioxidant capacity of 11S.

### 3.8. Surface Hydrophobicity of F11S

Hydrophobic interactions play a crucial role in protein stability, conformation, and functional properties, also influencing protein affinity for oil-water interfaces. 8-Anilino-1-naphthalenesulfonic acid (ANS) is a widely employed fluorescent probe for determining surface hydrophobicity, thereby assessing the exposure of hydrophobic regions within proteins [40]. The mechanism of ANS fluorescence involves weak emission in aqueous solutions. But upon binding to a protein’s hydrophobic cluster, its fluorescence intensity markedly increases, accompanied by a spectral λmax shift towards shorter wavelengths (blue shift).

**Figure 8:**
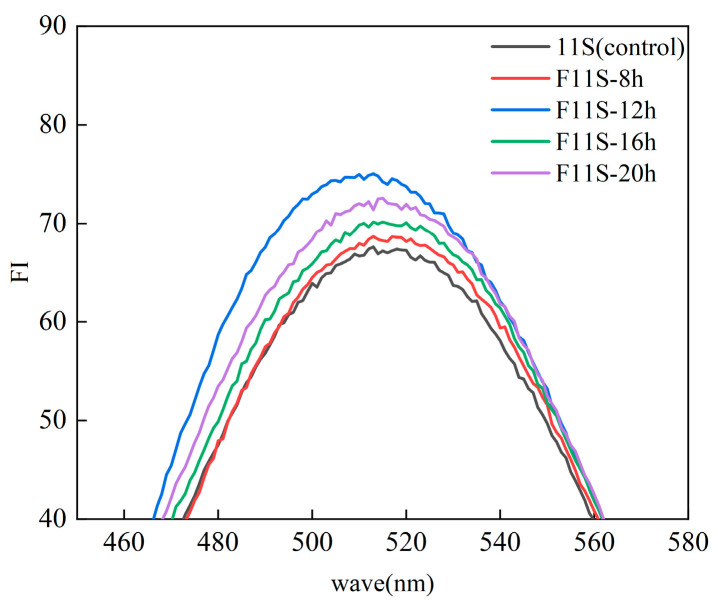
The ANS fluorescence intensity of F11S.

As shown in Figure 8, LAB fermentation led to a significant increase in the ANS fluorescence intensity of 11S, accompanied by a blue shift. This indicates that the fermentation process induces partial unfolding of the protein, leading to the exposure of hydrophobic regions and amino acid residues that were previously buried. Consequently, ANS binds to these newly accessible hydrophobic clusters, resulting in the observed enhanced fluorescence. Increased surface hydrophobicity may also enhance the protein’s ability to interact with reactive oxygen species (ROS), which are key players in lipid oxidation processes. Thus, the exposure of hydrophobic regions not only alters the protein’s structural stability but also contributes to its improved anti-lipid oxidation activity.

This structural rearrangement is characteristic of the molten-globule state, an intermediate conformation that represents a gradual transition between native and complete denaturation states. The molten-globule state retains secondary structures and intrinsic viscosities similar to the native state but lacks the specific tertiary structures formed by side chains. Furthermore, the protein molecule’s radius in this state can increase by 10–30% compared to its native conformation, and the abundant loosely packed hydrophobic core results in a larger accessible hydrophobic surface area [12]. A gradual increase in intensity between 8 and 12 h, reaching a peak at 12 h. Subsequently, the intensity exhibited varying degrees of attenuation at 16 and 20 h. Integrating these dynamic changes in ANS fluorescence intensity with the protein structural analyses presented in Section 3.6 and Section 3.7, we conclude that fermentation induces the formation of a molten globule state in soybean 11S protein.

It is acknowledged that LAB fermentation involves limited proteolysis, inevitably generating bioactive peptides. However, the superior emulsion stability of F11S (Figure 4 and Figure 5) strongly suggests the presence of substantial macromolecular structures rather than complete degradation into small peptides, as low-molecular-weight hydrolysates typically lack the viscoelasticity required to stabilize interfaces. Therefore, the enhanced functionality observed herein is attributed to a synergistic effect: the release of bioactive peptides and, crucially, the formation of the ‘molten globule-like’ state in the remaining protein macromolecules, which exposes functional groups while maintaining sufficient structural integrity for emulsification.

## 4. Conclusions

This study investigated the effects of lactic acid bacteria (LAB) fermentation on the structural modifications and antioxidant properties of soybean 11S protein (F11S). The in vitro antioxidant capacity of F11S was evaluated by measuring its lipid peroxidation inhibition, hydroxyl radical (·OH) scavenging ability, and DPPH radical scavenging activity across different fermentation durations. The results demonstrated that LAB fermentation significantly enhanced the antioxidant capacity of F11S. Specifically, at 8 h and 16 h of fermentation, the lipid peroxidation inhibition percentages of F11S increased to 37.75 ± 1.03% and 35.06 ± 0.4%, respectively. The highest ·OH and DPPH radical scavenging capacities (84.51 ± 2.53%) were observed at 16 h of fermentation.

To assess the anti-lipid thermal oxidation capacity of F11S, accelerated oil storage tests were conducted using the Schaal oven method, with peroxide value (POV) and acid value (AV) as indicators. When F11S was incorporated into pre-formed Tween 20 emulsions (added post-homogenization), significant inhibition of peroxide formation (*p* < 0.05) was observed in emulsions containing F11S fermented for 8–20 h after 9 days of storage. Notably, the F11S fermented for 16 h (F11-16 h) exhibited the lowest POV (4.33 ± 0.53 mmol/kg) after 15 days of storage, indicating superior anti-lipid thermal oxidation performance.

Structural analysis revealed that fermentation induced dynamic conformational changes in F11S, transitioning it into a molten globule-like state. The protein refolded into a more ordered conformation during fermentation, reaching maximum structural stability at 16 h. F11S fermented for 12 h displayed the strongest hydrophobic properties, while the compact molten globule structure of post-fermentation F11S contributed to its enhanced antioxidant capacity.

Meanwhile, the microbial fermentation process, especially through the action of LAB strains LF-56 and LF-57, plays a critical role in generating protein hydrolysates. The enzymes secreted by LAB are known for their heterogeneity and ability to break down 11S into bioactive peptides with improved antioxidant properties. This enzymatic degradation, driven by microbial fermentation, significantly enhances the functionality of the resulting peptides, supporting their increased antioxidant activity and lipid oxidation resistance. It is worth noting that metabolic by-products (e.g., organic acids, exopolysaccharides) generated by LAB may also contribute to the overall antioxidant capacity. However, the accumulation of these metabolites typically increases continuously with time. In contrast, the bioactivity of F11S in this study exhibited a ‘peak-and-decline’ trend (peaking at 16 h), which closely mirrors the structural evolution data (refolding and aggregation dynamics) rather than linear metabolite accumulation. This correlation suggests that the conformational state of the F11S plays a pivotal role in regulating its antioxidant potential.

These findings suggest that LAB fermentation optimizes the functional properties of soybean 11S protein by modulating the conformational state and hydrophobicity of the protein fraction, in concert with the generation of fermentation-derived peptides and metabolites, thereby improving their overall antioxidant and anti-lipid oxidation activities. Future research should explore the effects of LAB fermentation on other soy protein fractions, such as soy protein isolate (SPI) and 7S protein, to comprehensively evaluate their antioxidant potential. Additionally, the influence of different LAB strains on the antioxidant capacity of soy proteins warrants further investigation to identify optimal fermentation conditions for industrial applications. Furthermore, future studies should focus on elucidating the mechanisms through which protein hydrolysates generated during fermentation enhance antioxidant capacity, particularly how these peptides interact with reactive oxygen species (ROS) and modulate oxidative stress at the molecular level. This deeper mechanistic understanding will provide valuable insights into the functional enhancement of soy proteins and their potential applications in food and health industries.

## Data Availability

The original contributions presented in this study are included in the article. Further inquiries can be directed to the corresponding authors.
